# Detection of *Mycobacterium tuberculosis* Complex in Sputum Samples Using Droplet Digital PCR Targeting *mpt64*

**DOI:** 10.3390/pathogens12020345

**Published:** 2023-02-18

**Authors:** Ye Win Aung, Kiatichai Faksri, Arunnee Sangka, Kanchana Tomanakan, Wises Namwat

**Affiliations:** 1 Department of Microbiology, Faculty of Medicine, Khon Kaen University, Khon Kaen 40002, Thailand; 2 Faculty of Associated Medical Science, Khon Kaen University, Khon Kaen 40002, Thailand; 3 Department of Medical laboratory, Khon Kaen Hospital, Khon Kaen 40000, Thailand

**Keywords:** droplet digital PCR, GeneXpert MTB/RIF assay, leftover sputum, *mpt64* gene, tuberculosis

## Abstract

Tuberculosis (TB) is one of the top 10 causes of death worldwide. It is challenging to find methods of diagnosis of active pulmonary TB that are sensitive enough to detect cases for proper treatment before unintentional transmission. Droplet digital PCR (ddPCR) is a highly sensitive method to detect genetic material of pathogens, but it has rarely been used for diagnosis of TB. This study compared the sensitivity of ddPCR with that of GeneXpert and AFB smear microscopy in 180 leftover sputum samples from patients suspected of having TB on the basis of clinical symptoms and radiography. Absolute quantification of copy numbers of MTB-specific genes was possible using ddPCR targeting the *mpt64* gene. Among the 180 samples, 41.1% were diagnosed as having TB using ddPCR. The sensitivities of AFB smear microscopy, GeneXpert and ddPCR were 41.9%, 82.4% and 100%, respectively. AFB smear microscopy and GeneXpert both had a specificity of 100%, and the specificity of ddPCR was 95.3%. The accuracy of ddPCR (97.2%) is higher than that of GeneXpert (92.7%). This robust ddPCR system could potentially be used as a method for early diagnosis of TB.

## 1. Introduction

Tuberculosis (TB) is a chronic infectious disease and a major public health disease caused by the bacterium *Mycobacterium tuberculosis* (MTB). In 2017 alone, there were an estimated 10 million new cases and 1.3 million deaths due to the disease. Thus, it is one of the top 10 causes of death worldwide. In Thailand, 108,000 people were newly infected and 9300 people died due to tuberculosis in 2017. Thailand ranks 26th among the top 30 high TB-burden countries [1]. Early TB diagnosis and effective treatment are crucial to control the disease and reduce its incidence [2].

Diagnostic methods such as acid-fast bacilli (AFB) smear microscopy are simple and sensitive, but do not accurately indicate the number of AFB in the samples, information that is required for monitoring of anti-TB treatment. Many molecular methods have been used to in an effort to achieve early TB diagnosis. Prominent among these are nucleic acid amplification tests [3]. On the other hand, molecular methods targeting specific genes have the potential to quantify AFB in a sample and provide a means of monitoring tuberculosis treatment. Digital PCR (dPCR) is one such method [4]. Copy numbers of the genes of the *Mycobacterium tuberculosis* complex (MTBC) have been quantified using dPCR assays for target genes such as *16S rRNA* and *rpoB* [4], *IS6110* [5,6] and *gyrB* [6] assays of ddPCR and *mpt64* assays using real-time PCR [7,8,9]. In clinical diagnosis, many patients have been diagnosed as having active TB based only on clinical symptoms and chest X-ray, even if they are AFB-negative or pathogen nucleic acid cannot be detected [10]. A more effective method to assess these problematic cases is needed.

Droplet digital PCR (ddPCR) belongs to the third generation of PCR technologies and is based on a water–oil emulsion droplet system. This system uses a combination of chemical surfactants and microfluidics to partition the molecular samples into water droplets within an oil emulsion. The droplet generator machine converts 20 µL of aqueous sample containing template DNA into 20,000 nanoliter-sized droplets, within each of which a PCR reaction takes place. While partitioning the sample, target DNA are discretely found in the droplets covered by oil. Thus, a ddPCR system can easily conduct many thousands of discrete reactions. After PCR amplification, the droplet reader (flow cytometry) analyzes each droplet to enumerate those positive or negative for template amplification [11]. Droplet digital PCR can detect the copy number of target DNA in small volumes of nucleic acid without a standard curve [12]. Additional advantages are absolute quantification, unparalleled precision, superior partitioning and the best accuracy in the presence of PCR inhibitors [13]. This technology has rarely been used for TB diagnosis [14] and its performance has never been investigated in a country with a high TB burden. 

The Mycobacterium Protein Tuberculosis (*mpt64*) or Mannose Binding Protein 64 (*mpb*64) gene is a protein weighing 24 kDa that is one of the genomes of the cell wall. This protein is produced during the replication of MTB and is absent in atypical mycobacteria [15]. It is also an immunogenic protein and a protein-coding gene. The length of this gene is 687 base pairs [16]. The encoding *mpt64* gene, one of the proteins secreted by MTBC, has been used for diagnosis of TB [7]. Molecular diagnosis by RT-PCR using *mpt64* assay demonstrated high sensitivity, specificity and accuracy for early detection of TB in extra-pulmonary [7] and pulmonary TB [7,8]. The major MTB-specific genes, including *IS6110*, *16S rRNA* and *mpt64* genes, were used to detect the targeted DNA of MTB via PCR [17,18]. Among these genes, *IS6110* and *16S rRNA* genes have been frequently applied as the targeted sequences of the DNA of MTB for PCR in many clinical laboratories [18,19]. Advantages of *IS6110* usage for the detection of MTBC are a repetitive mobile specific sequence for MTB genome, a fast diagnostic target, higher sensitivity and usage for many years to diagnose and genotype this pathogen [20]. Disadvantages of its usage are: the variable number of *IS6110* copies are increased in different strains due to the insertion in a transcriptionally active region of the genome, leading to the removal of some parts of the genome [21]. There are some strains of MTB that lack the *IS6110* sequence in some parts of Southeast Asia [22]. 

Benefits of *16S rRNA* usage for the detection of MTBC are: This gene is universally present in prokaryotes, and it is rarely affected by horizontal gene transfer. It contains several hyper-variable regions, allowing for the distinction of different prokaryotes, leading to the fabrication of universal primer designs that can be used to amplify *16S rRNA* [23]. The drawbacks of its usage are: it has a varying number of copies in approximately 80% of all fully sequenced genomes in prokaryotes and can only identify to the genus level due to high similarity between *16S rRNA* genes from closely related species [24]. The specific primers and TaqMan probes for the *mpt64* gene were reported to be the most sensitive and specific sequence of the MTB DNA for the PCR assay [25,26]. Strengths of *mpt64* gene usage for MTBC detection are: this gene is a highly conserved secretory protein, harboring only a few mutations, and it is used in rapid diagnosis of MTBC in high TB-burden countries. The weaknesses of its usage are: discrepant results are found due to mutations in the *mpt64* gene when comparing with the results of microscopy, culture and GeneXpert techniques [27]. The nonspecifically positive result of *mpt64* assay is also seen in *Mycobacterium scrofulaceum* [28].

This study aimed to evaluate and measure the performance of ddPCR targeting the *mpt64* gene for direct detection of members of the MTBC in sputum samples from patients who had been clinically diagnosed as having active pulmonary TB. This would clarify whether the absence of AFB in these samples represented false negatives. Using ddPCR, we hoped to detect the absolute number of MTB cells in the samples, leading to early diagnosis and initiation of anti-TB. There has been no previous evaluation of ddPCR targeting *mpt64* for the detection of members of the MTBC.

## 2. Materials and Methods

### 2.1. Sample Collection and Sample Size Calculation

The samples came from suspected TB cases (n = 180). This diagnosis was reached using clinical symptoms, including chronic cough for more than 3 weeks, cough with sputum or blood, low-grade fever, weight loss, chest pain and tiredness and abnormal chest X-rays. These suspected cases included 31 that were AFB-positive and 61 positives according to GeneXpert. All were recruited from Khon Kaen Hospital, Khon Kaen, Northeast Thailand. The sputum samples were collected in wide-mouth, screw-cap, no-leakage containers and put into biohazard-labeled plastic bags. The following demographic data were provided by Khon Kaen Hospital: sample ID, age and sex of patient, province of residence, type of sample, AFB grading, type of TB patient, results of GeneXpert MTB/RIF assay and TB diagnosis that was finally reached. The final diagnosis was confirmed using the results of AFB smear microscopy, GeneXpert MTB/RIF assay, clinical symptoms and radiological findings. The sample size used in this study is sufficient according to our sample-size calculation (96 samples required).

### 2.2. Sputum Samples Processing

The leftover sputum samples (2.5 mL) from Khon Kaen Hospital were first decontaminated with an equal amount of 1% N-acetyl L-cysteine sodium hydroxide (1% NALC-NaOH). The supernatants were slowly discarded into a disinfectant bottle. The sediments were dissolved again with distilled water and used for DNA extraction to detect members of the MTBC using ddPCR [29].

### 2.3. Nucleic Acid Extraction 

Briefly, 900 µL of bacterial suspension was transferred to a 1.5 mL microcentrifuge tube and then was incubated at 80 °C for 40 min to inactivate the bacteria [30]. The suspension was centrifuged at 8000 rpm for 1 min. Then, the supernatants were carefully discarded and the bacterial pellets were collected for DNA extraction. Genomic DNA was extracted and purified by using a QIAamp DNA Mini kit (QIAGEN, Hilden, Germany) according to the manufacturer’s instructions. The concentration of purified DNA was measured using a NanoDrop 2000c Spectrophotometer (Thermo scientific^®^, Waltham, MA, USA).

### 2.4. Primers and Probe for mpt64 Gene

The primers to amplify *mpt64* for MTBC detection were those described previously [26], forward: GTGAACTGAGCAAGCAGACCG, and reverse: GTTCTGATAATTCACCGG GTCC. The probe used was FAM-TATCGATAGCGCCGAATGCCGG-Iowa Black. The target DNA products were detected using a TaqMan probe labeled with “FAM” fluorophore at 5′ end and “Iowa Black” quencher at the 3′ end. We analyzed and checked In-Silico PCR of the conservative primers for *mpt64* gene in the UCSC Genome Browser on ASM19595v2 February 2013, *Mycobacterium tuberculosis* (H37Rv 2013) (GCF_000195955.2), at https://genome.ucsc.edu/cgi-bin/hgPcr (accessed on 7 February 2023). As a result, we obtained the 77 bp amplicon sequence (NC_000962.3:2223463-2223539) matched with MTB (H37Rv, 2013). Therefore, this primer and probe design for *mpt64* is still suitable for the detection of MTBC.

### 2.5. ddPCR System and Conditions

The present study used a ddPCR system including a QX200 Droplet Generator (Bio-Rad, Hercules, CA, USA), T100 Thermal Cycler PCR (Bio-Rad, Hercules, CA, USA) and a QX200 Droplet Reader (Bio-Rad, Hercules, CA, USA). The total volume of the ddPCR reaction was 20 µL, consisting of 10 µL of 2x ddPCR supermix for the probe (Bio-Rad, Hercules, CA, USA), 1 µL of 20x target (FAM) primers and probe for *mpt64* (Bio-Rad, Hercules, CA, USA), 1 µL of Hind III (HF) (New England BioLabs, Hitchin, UK), 5 µL of nuclease-free water and 3 µL of purified DNA sample. The ddPCR reaction mixtures and the generation oil for the probe (Bio-Rad, Hercules, CA, USA) were separately transferred to the eight wells of DG8 cartridges (Bio-Rad, Hercules, CA, USA), then were loaded into a QX 200 Droplet Generator to produce nanoliter-sized droplets. These were transferred to a 96-well PCR plate (Bio-Rad, Hercules, CA, USA) which was sealed with a heat-seal foil (Bio-Rad, Hercules, CA, USA) in a PX1TM PCR plate sealer (Bio-Rad, Hercules, CA, USA) at 180 °C for 5 s. The plate was transferred into a T100 Thermal Cycler run using the following cycle conditions: the enzyme activation step at 95 °C for 10 min, followed by 40 cycles consisting of a denaturation step at 94 °C for 30 s, an annealing step at 51.9 °C for 1 min and a final step at 98 °C for 10 min. The temperature ramp rate was 2 °C/s. The best annealing temperature was 51.9 °C, which was selected from the optimization of a temperature gradient between 50 °C and 60 °C. All tests of each sample were performed in duplicate. MTB H37Rv DNA, *Mycobacterium abscessus* DNA and nuclease-free water were used as the positive control, negative control and non-template control (NTC), respectively.

### 2.6. Lower Limit of Detection (LLD)

The lower limit of detection (LLD) for *mpt64* assay was performed in duplicate by using the extracted and purified H37Rv DNA. The MTB DNA was diluted in nuclease-free water to make serial 10-fold dilutions from 1 ng/µL until 10^−6^ dilution or 1 fg/µL concentration of MTB DNA. 

### 2.7. Data Analyses

After the amplification process, the 96-well PCR plates were loaded into a QX 200 Droplet Reader. The reader measured the fluorescence intensity signals within the amplified droplets using QuantaSoft^TM^ software version 1.7.4 (Bio-Rad, Hercules, CA, USA). This software was used for interpreting and counting the number of positive and negative droplets per fluorophore per sample. The formula used by QuantaSoft^TM^ software to calculate the concentration (copies per microliter; CPM in reaction mixture of ddPCR) was the following [31];

Concentration = −In (N_neg_/N)/V_droplet_

where In = Inputting n, N_neg_ = Number of negative droplets, N = Total number of droplets, and Vdroplet = Volume of droplets. The DNA concentration in the sputum was calculated using the following formula:

Total copies = (CPM × V_reaction_/V_used DNA_) × V_used DNA_ × (C_total DNA_/C_used DNA_)

where CPM = CPM in the reaction mixture, V_reaction_ = volume of the total reaction, V_used DNA_ = volume of the used DNA, C_total DNA_ = concentration of the total DNA and C_used DNA_ = concentration of the used DNA. To calculate CPM in the sputum samples, the following equation was used:

CPM in sputum = Total copies in sputum/Volume of sputum

After a quality check, the software automatically set up the threshold level; the droplets above the level were considered as positive and those below were assumed to be negative. The fluorescence intensities for the ideal droplets determined the threshold levels for differentiating the positive and negative droplets by applying the k-nearest neighbor algorithm, “define the rain” [32]. The patient data from Khon Kaen Hospital were analyzed for any correlation with the results of ddPCR using IBM SPSS Statistics software, version 20 (IBM Corp, Armonk, NY, USA) and GraphPad prism 5 software (GraphPad, San Diego, CA, USA), which was used to create graphical charts. A Mann–Whitney test was used to find significant differences between different groups. The significance *p* value ≤ 0.05 was considered to be statistically significant, where * denotes 0.05, ** denotes 0.01; and *** denotes 0.001; ns: not significant. The sensitivity and specificity of ddPCR were calculated using true positive (TP) divided by TP plus false negative (FN) and true negative (TN) divided by TN plus false positive (FP), respectively, based on final diagnosis of the suspected TB patients.

## 3. Results

We used samples from 180 clinically suspected pulmonary TB cases. The TB diagnosis was based on AFB smear microscopy, GeneXpert MTB/RIF assay, clinical symptoms and chest X-ray. Of the samples, 74 (41.1%) were diagnosed as TB and the remaining 106 (58.9%) out of 180 samples were non-TB cases. The average age of suspected TB patients was 57.5 ± 17.2 years. The highest and second-highest suspected TB age groups were 41–60 years old (30, 40.5%) and 61–80 years old (24, 32.4%). There were 62 female patients (34.4%) and 118 male patients (65.6%). Most of the suspected patients, 175 (97.1%), were from Khon Kaen Province. The suspected TB patients were categorized into three groups, that is, non-TB patients (106, 58.9%), new TB patients (71, 39.4%) and relapsed TB patients (3, 1.7%) (Table 1).

**Figure 1:**
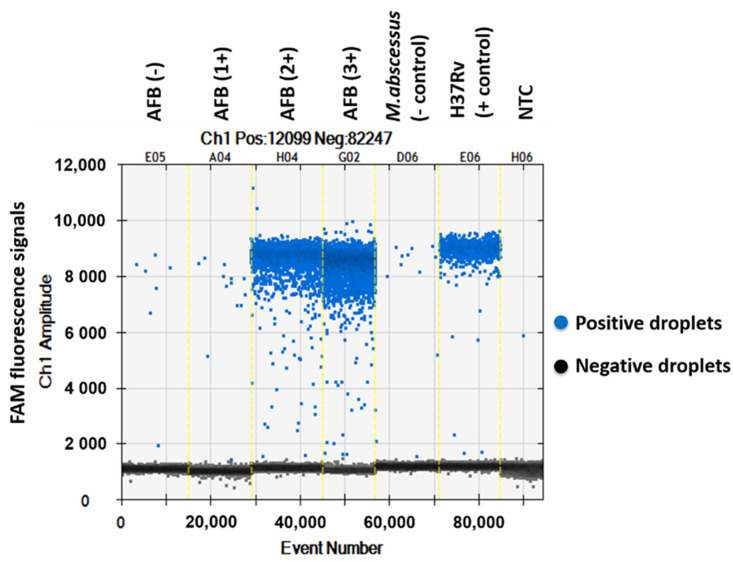
One-dimensional amplitude of FAM channel detecting the specific DNA of MTBC in sputum samples via ddPCR using *mpt64* gene primers and probe.

**Figure 2:**
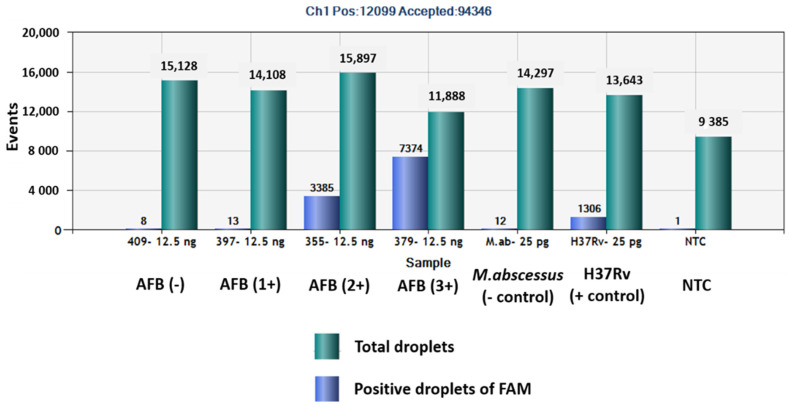
Total numbers of positive droplets containing specific DNA according to the FAM channel for detection of MTBC by ddPCR using *mpt64* gene primers and probe.

The QuantaSoft^TM^ software measured the FAM fluorescence signals within the nanoliter-sized droplets to detect the specific DNA of MTBC in the sputum samples. Positive droplets were shown above the threshold and negative droplets were revealed below the threshold level. The software counted the total number of positive droplets containing target DNA according to the FAM channel for detection of the MTBC. The copy numbers of *mpt64* in samples classed as AFB (-), AFB (1+), AFB (2+), AFB (3+), negative control, positive control and NTC were 0.6, 2.2, 282, 1139, 1.9, 118 and 0.1 copies/µL, respectively (Figure 1 and Figure 2).

In this study, the copy numbers for the LLD of *mpt64* assay were found to be 1 ng, 0.1 ng, 10 pg, 1 pg, 0.1 pg, 10 fg, 1 fg and those of the negative control were 217,391.3, 21,739.1, 2173.9, 217.4, 21.7, 2.17, 0.2 and 1.9 copies/µL in the reaction mixture, respectively. The LLD for the *mpt64* assay was determined to be 10 fg (2.17 copies/µL) based on the comparison of copy numbers between the test samples and the negative control (*Mycobacterium abscessus*). The results of the study show that out of 180 samples, 101 (56.1%) were negative in ddPCR reaction, AFB smear microscopy and GeneXpert MTB/RIF assay and had copy numbers ranging from 0.1 to 1.0 copies/µL in the reaction mixture. When deciding the cut-off value for identifying negative and positive samples, it is important to consider the LLD of the assay and the range of copy numbers in the negative samples. In this case, a cut-off value of 2.17 copies/µL, which corresponds to the LLD of 10 fg, could be considered as a reasonable threshold for identifying positive samples.

**Figure 3:**
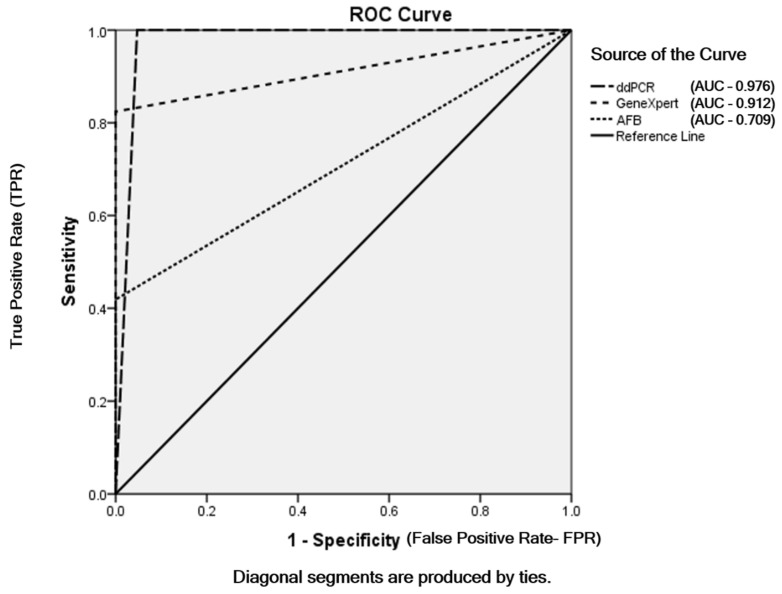
ROC curve of AFB smear microscopy, GeneXpert MTB/RIF assay and ddPCR depending on final TB diagnosis.

Among the 180 samples, 31 (17.2%) were AFB smear positive and 149 (82.8%) were AFB-negative. The MTBC detected samples using GeneXpert MTB/RIF assay were 61 (33.9%) and MTBC not-detected samples were 119 (66.1%) out of total patients. According to the *mpt64* assay using ddPCR, 79/180 patients (43.9%) were infected with members of the MTBC and 101/180 (56.1%) were not. Out of the 79 MTBC samples detected using ddPCR, 5 were false positives. The sensitivities of AFB smear microscopy, GeneXpert MTB/RIF assay and ddPCR were 41.9%, 82.4% and 100%, respectively. AFB smear microscopy and the GeneXpert MTB/RIF assay had the same specificity (100%), and the specificity of ddPCR was 95.3%. The same positive predictive value (PPV) 100% was found in AFB smear microscopy and GeneXpert and the PPV of ddPCR was 93.7%. The negative predictive values (NPV) of AFB smear microscopy, GeneXpert and ddPCR were 71.1%, 89.1% and 100%, respectively (Table 2). The accuracies of AFB smear microscopy, GeneXpert and ddPCR were 76.1%, 92.7% and 97.2%, respectively. The sensitivity and specificity for AFB smear microscopy, GeneXpert MTB/RIF assay and ddPCR were based on the final TB diagnosis shown in ROC curve (Figure 3).

**Figure 4:**
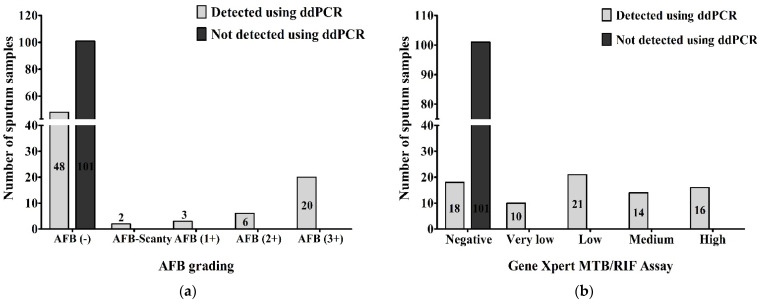
Number of MTBC detected and not-detected sputum samples compared between (**a**) AFB grading and ddPCR; (**b**) GeneXpert MTB/RIF assay and ddPCR.

When we compared the results of AFB grading and ddPCR, we found that 48 (26.7%) of AFB-negative, 2 (1.1%) of AFB-Scanty, 3 (1.7%) of AFB (1+), 6 (3.3%) of AFB (2+) and 20 (11.1%) of AFB (3+) were detected as MTBC and 101 (56.1%) of AFB-negative out of a total of 180 samples were not detected as MTBC using ddPCR. Using droplet digital PCR, we detected MTBC in all AFB-positive patients, 31 (17.2%) of the total samples (Figure 4a). Comparing between the results of GeneXpert MTB/RIF assay and ddPCR, 18 (10%) of GeneXpert-negative, 10 (5.5%) of very low concentration, 21 (11.7%) of low concentration, 14 (7.8%) of medium concentration and 16 (8.9%) of high concentration of MTBC in GeneXpert out of total 180 samples were detected as MTBC using ddPCR. Using droplet digital PCR, we did not detect MTBC in 101 (56.1%) of GeneXpert-negatives out of the total samples. All GeneXpert-positive samples, 61 (33.9%) of the total samples, were found as MTBC via ddPCR (Figure 4b).

**Figure 5:**
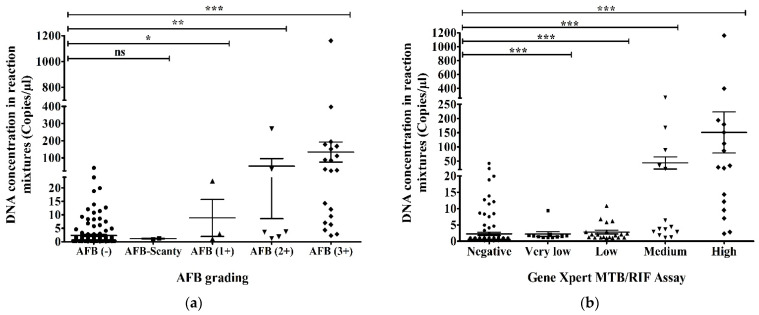
DNA concentration of MTBC in reaction mixture (copies/µL) using ddPCR compared to (**a**) AFB grading and (**b**) GeneXpert MTB/RIF assay. The statistical significance was assessed using a Mann–Whitney test between groups; *p* values ≤ 0.05 were considered to be statistically significant where * denotes 0.05, ** denotes 0.01; and *** denotes 0.001; ns: not significant. Error bars represent mean ± standard error of mean (SEM). SEM is calculated by dividing the standard deviation (SD) by the square root of the count (N).

The highest (1160.5 copies/µL in reaction mixture) and the second-highest (396.5 copies/µL in reaction mixture) DNA concentrations according to ddPCR were found in AFB (3+) samples and high concentration samples using GeneXpert. There were 48 AFB-negative and ddPCR-positive samples and the range of their DNA concentration in the ddPCR reaction mixture was 2.2–41.9 copies/µL (Figure 5a). We found that 18 samples were GeneXpert-negative and ddPCR-positive samples. Their DNA concentration was revealed to be 2.2–41.9 copies/µL in the ddPCR reaction mixture (Figure 5b). AFB-negative and GeneXpert-negative samples had the lowest DNA concentration (0.1 copies/µL) in the reaction mixture of ddPCR (Figure 5a,b).

We calculated the DNA concentration in the sputum according to the following formulas:

$$\begin{array}{ll} \mathrm{Total}\;\mathrm{copies} & =(\mathrm{CPM}\times V_{\mathrm{reaction}}/V_{\mathrm{used}\;\mathrm{DNA}})\times V_{\mathrm{used}\;\mathrm{DNA}}\times (C_{\mathrm{total}\;\mathrm{DNA}}/C_{\mathrm{used}\;\mathrm{DNA}})\\ & =(168\;\mathrm{copies}/µL\times 20\;µL/3\;µL)\times 3\;µL\times (680\;\mathrm{ng}/12.5\;\mathrm{ng})\\ & =182,784\;\mathrm{copies} \end{array}$$

$$\begin{array}{ll} \mathrm{CPM}\;\mathrm{in}\;\mathrm{sputum} & =\mathrm{Total}\;\mathrm{copies}\;\mathrm{in}\;\mathrm{sputum}/\mathrm{Volume}\;\mathrm{of}\;\mathrm{sputum}\\ & =182,784\;\mathrm{copies}/2500\;µL\\ & =73.11\;\mathrm{copies}/µL \end{array}$$

**Figure 6:**
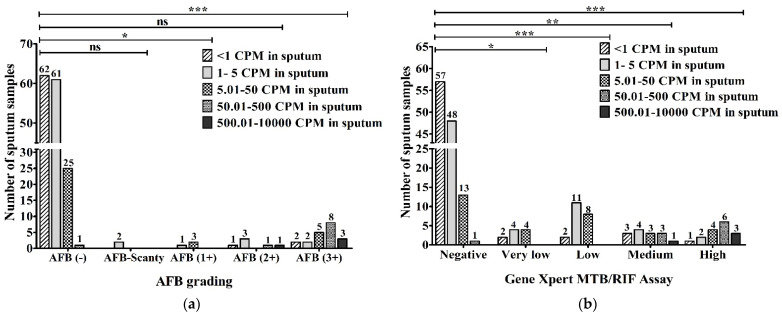
Evaluation between (**a**) AFB grading (AFB/µL); (**b**) GeneXpert MTB/RIF assay and DNA concentration in sputum (copies/µL) via ddPCR (CPM-copies per microliter). The statistical significance was assessed using a Mann–Whitney test between groups; *p* values ≤ 0.05 were considered to be statistically significant where * denotes 0.05, ** denotes 0.01; and *** denotes 0.001; ns: not significant.

The precise numbers of MTBC in the sputum obtained via ddPCR were evaluated and compared with AFB grading. Out of 149 AFB-negative samples, 62 (34.4%) had <1 copies per microliter (CPM), 61 (33.8%) contained 1–5 CPM, 25 (13.8%) had 5.01–50 CPM and 2 (1.1%) had 50.01–500 CPM in the sputum according to ddPCR. Two (100%) of two AFB-Scanty (5 AFB/µL) samples had 1–5 CPM in sputum. Among three AFB (1+) (50 AFB/µL) samples, one (33.3%) was detected to have 1–5 CPM and two (66.7%) were found to have 5.01–50 CPM in their sputum. In total, out of six AFB (2+) (500 AFB/µL) samples, one (16.7%) sample was found for <1 CPM, 50.01–500 CPM and 500.01–10,000 CPM each and three (50%) were detected to have 1–5 CPM in the sputum. Out of a total of 20 AFB (3+) (5000 AFB/µL), 2 (10%) samples were found in the <1 CPM group as well as the 1–5 CPM group, 5 (25%) samples were detected to have 5.01–50 CPM, 8 (40%) samples were found to have 50.01–500 CPM, and 3 (15%) samples were found to have 500.01–10,000 CPM in the sputum using ddPCR (Figure 6a).

When the DNA concentrations in sputum (copies/µL) using ddPCR were compared with the results of the GeneXpert MTB/RIF assay, 57 (47.9%), 48 (40.4%), 13 (10.9%) and 1 (0.8%) of a total of 119 GeneXpert-negative samples had <1, 1–5, 5.01–50 and 50.01–500 CPM, respectively. Out of 10 samples with very low MTB-concentration, 2 (20%), 4 (40%) and another 4 (40%) samples had <1, 1–5 and 5.01–50 CPM, respectively. In 2 (9.5%), 11 (52.4%) and 8 (38.1%) of 21 low MTB-concentration samples, we found <1, 1–5 and 5.01–50 CPM, respectively. Among a total of 14 medium MTB-concentration samples, 4 (28.6%) had 1–5 CPM, 1 (7.2%) sample had 500.01–10,000 CPM, and there were 3 (21.4%) in each group of <1 CPM, 5.01–50 CPM and 50.01–500 CPM. Out of a total of 16 high MTB-concentration samples, 1 (6.3%), 2 (12.5%), 4 (25%), 6 (37.5%) and 3 (18.7%) were detected to have <1, 1–5, 5.01–50, 50.01–500 and 500.01–10,000 CPM, respectively, in the sputum via ddPCR (Figure 6b).

## 4. Discussion

In the present study, 41.1% of the samples were diagnosed with TB and 58.9% were non-TB cases. Previous studies found that the confirmed TB and non-TB cases were 8.7% and 91.3% [8] and 26.7% and 73.3% [33], respectively. The accuracy of ddPCR, AFB smear microscopy and GeneXpert were 97.2%, 76.1% and 92.7%, respectively, in this study. ddPCR is higher accuracy than conventional PCR due to its ability to divide the sample into nano-droplets, resulting in a high number of partitions per reaction and improved accuracy in detecting low concentrations of the target DNA [34]. In our study, the sensitivity and specificity of the *mpt64* assay in ddPCR were 100% and 95.3%, respectively. The sensitivity and specificity of the *mpt64* assay using RT-PCR to detect MTB from the peripheral blood were 30.8% and 88.8%, respectively [35]. ddPCR is a highly sensitive and specific method for nucleic acid quantification that has no need for a calibration curve [14]. The sensitivity and specificity of the *mpt64* antigen test were 92% and 95%, respectively [36]. 

In this study, the sensitivity and specificity of AFB smear microscopy and GeneXpert were 41.9% and 100% and 82.4% and 100%, respectively. A previous study revealed that the sensitivity and specificity of AFB smear microscopy were 22.2% and 78.5%, respectively, and the overall sensitivity and specificity of GeneXpert were 86.8% and 93.1%,respectively [37]. The study found that 56.1% of the samples were negative in ddPCR, AFB smear microscopy and GeneXpert MTB/RIF assay. In the present study, the false positives of the *mpt64* assay using ddPCR were 2.8%, and the false negatives were 0%. Although there were five false positive patients, ddPCR detected high *mpt64* copy numbers in reaction mixtures from these patients (8.3, 8.6, 11.4, 18.8, 23.9 copies/µL). Among them, two patients were expired, one with lung abscess and one with unknown diagnosis, and another two patients experienced remission of clinical signs and symptoms of lung abscess; the last patient, diagnosed only as empyema, was lost upon follow up. The false positives and false negatives of the *mpt64* assay using RT-PCR were 1.3% and 0.8% [8], respectively, and another study found that false positives and false negatives of *mpt64* assay in RT-PCR were 1.4% and 60.8%, respectively [35].

AFB-smear microscopy has a lower limit of detectability of about 5000–10000 AFB/mL in the sputum [38]. So, the AFB-Scanty, AFB (1+), AFB (2+) and AFB (3+) categories should indicate the presence of 5 AFB/µL, 50 AFB/µL, 500 AFB/µL and 5000 AFB/µL in the sputum, respectively. The present study found that 41.6% of AFB-negative, 100% of AFB-Scanty, 33.3% of AFB (1+), 33.3% of AFB (2+) and 15% of AFB (3+) samples had similar numerical ranges of MTB cells in the sputum compared to ddPCR. A previous study comparing detection of the MTBC via AFB smear and qPCR using a *IS6110* assay found that 65.4% of AFB-negative, 36.4% of AFB-Trace, 36% of AFB (1+), 0% of AFB (2+) and 7.7% of AFB (3+) samples all had the same numbers of bacteria [39]. This study aimed to evaluate the performance of ddPCR in detecting the presence of MTBC in sputum samples. It was able to detect the MTBC in a high percentage of samples, including 100% of AFB- and GeneXpert-positive samples and in some AFB- and GeneXpert-negative samples at 32% and 15%, respectively. However, ddPCR also had some limitations, such as a long turnaround time, high cost per reaction, and the need for well-trained personnel. ddPCR performed reliably and accurately, as every case that was positive according to use of AFB-smear microscopy and GeneXpert was also positive according to use of ddPCR. Moreover, use of ddPCR was able to detect the presence of the MTBC in some samples that were negative according to use of AFB-smear microscopy and GeneXpert. 

## 5. Conclusions

We conclude that use of ddPCR rapidly and precisely evaluated the numbers of MTBC cells in the sputum. We demonstrated that use of ddPCR detected the MTBC in the sputum of all AFB- and GeneXpert-positive cases and in some negative cases. AFB-smear microscopy and GeneXpert did not find MTBC in some sputum samples, but ddPCR absolutely quantified the MTB DNA concentration in those samples. We found substantial deviations in results obtained using AFB-smear microscopy and GeneXpert from results obtained via ddPCR. The accuracy, sensitivity and specificity of ddPCR in the present study was higher than those of other parameters, such as AFB smear microscopy and GeneXpert MTB/RIF assay. Our ddPCR assay has the potential to permit early diagnosis of TB, leading to prompt and effective anti-TB treatment. Consequently, we could control the transmission of tuberculosis to healthy persons and prevent the emerging of drug-resistant tuberculosis in the community. This is why the ddPCR system could possibly be used as a valuable tool for the early detection of MTBC in sputum samples. However, further research is needed to determine the optimal use of ddPCR in the diagnostic workflow for tuberculosis. 

## Figures and Tables

**Table 1:** Demographic data of patients providing samples and the diagnoses reached.

Characteristics	TB Diagnosis ^1^ n (%)	Non-TB Diagnosis ^2^ n (%)	Total, n (%)	*p* Value
Age (Mean ± SD)	53.6 ± 15.6	60.2 ± 17.7	57.5 ± 17.2	
Age group (in years)				0.050 *
≤20	2 (2.7)	2 (1.9)	4 (2.2)	
21–40	15 (20.3)	11 (10.4)	26 (14.4)	
41–60	30 (40.5)	34 (32.1)	64 (35.6)	
61–80	24 (32.4)	44 (41.5)	68 (37.8)	
≥80	3 (4.1)	15 (14.1)	18 (10.0)	
Gender				0.266
Female	22 (29.7)	40 (37.7)	62 (34.4)	
Male	52 (70.3)	66 (62.3)	118 (65.6)	
Province ^3^				0.292
Chaiyaphum	2 (2.7)	0 (0.0)	2 (1.1)	
Kalasin	0 (0.0)	1 (0.9)	1 (0.6)	
Khon Kaen	72 (97.3)	103 (97.3)	175 (97.1)	
Mukdahan	0 (0.0)	1 (0.9)	1 (0.6)	
Udon Thani	0 (0.0)	1 (0.9)	1 (0.6)	
Type of patient				0.000 ***
Non-TB	0 (0.0)	106 (100)	106 (58.9)	
New TB	71 (95.9)	0 (0.0)	71 (39.4)	
Relapse	3 (4.1)	0 (0.0)	3 (1.7)	

^1^: Final TB diagnosis was based on the results of AFB smear microscopy, GeneXpert MTB/RIF assay, clinical symptoms and radiological findings. ^2^: Non-TB diagnosis was found not to be TB from the suspected patients. ^3^: Province in Northeastern Thailand. Statistical significance was assessed using a Mann–Whitney test between groups; *p* values ≤ 0.05 were considered to be statistically significant, where * denotes 0.05, and *** denotes 0.001.

**Table 2:** Results of AFB smear microscopy, GeneXpert MTB/RIF assay and ddPCR according to final TB diagnosis.

Methods	TB * n (%)	Non-TB n (%)	Total n (%)	Sensitivity (%)	Specificity (%)	PPV (%)	NPV (%)
AFB smear microscopy				41.9	100	100	71.1
Positive	31 (41.9)	0 (0)	31 (17.2)				
Negative	43 (58.1)	106 (100)	149 (82.8)				
GeneXpert MTB/RIF assay				82.4	100	100	89.1
Detected	61 (82.4)	0 (0)	61 (33.9)				
Not detected	13 (17.6)	106 (100)	119 (66.1)				
ddPCR, *mpt64*				100	95.3	93.7	100
Detected	74 (100)	5 (4.7)	79 (43.9)				
Not detected	0 (0)	101 (95.3)	101 (56.1)				

*: Final TB diagnosis was based on the results of AFB smear microscopy, GeneXpert MTB/RIF as-say, clinical symptoms and radiological findings.

## Data Availability

Data are available in Table 1 and Table 2, Figure 1, Figure 2, Figure 3, Figure 4, Figure 5 and Figure 6.
